# Comparative Cochlear Transcriptomics in Echolocating Bats and Mouse Reveals Hras as Protector Against Noise‐Induced Hearing Loss

**DOI:** 10.1002/advs.202508466

**Published:** 2025-09-08

**Authors:** Peng Chen, Chenhao Che, Lingjie Wu, Changjie Sun, Dongming Xu, Qinyang Hua, Yunzhong Zhang, Yi‐Quan Tang, Peng Shi, Shan Sun

**Affiliations:** ^1^ ENT Institute and Otorhinolaryngology Department of Eye & ENT Hospital State Key Laboratory of Brain Function and Disorders and MOE Frontiers Center for Brain Science Fudan University Shanghai 200031 China; ^2^ Shanghai Key Laboratory of Gene Editing and Cell Therapy for Rare Diseases Fudan University Shanghai 20031 China; ^3^ Key Laboratory of Genetic Evolution & Animal Models Kunming Institute of Zoology Chinese Academy of Sciences Kunming 650223 China; ^4^ School of Future Technology University of Chinese Academy of Sciences Beijing 101408 China; ^5^ Kunming College of Life Science University of Chinese Academy of Sciences Beijing 101408 China; ^6^ Institutes of Brain Science Fudan University Shanghai 200032 China

**Keywords:** cochlear hair cells, echolocating bats, Hras, mouse, noise‐induced hearing loss

## Abstract

Noise‐induced hearing loss (NIHL), caused by irreversible cochlear hair cell (HC) damage, lacks effective therapies due to a limited understanding of endogenous protective mechanisms. The echolocating bats exhibit natural resistance to intense noise, and this suggested novel insights into methods to protect against NIHL. Here, through comparative transcriptomic analysis of noise‐exposed cochleae from the eastern bent‐winged bats (*Miniopterus fuliginosus*) and mice, the specific transcriptional dynamics in noise‐resistant *Miniopterus fuliginosus* are revealed, thus highlighting potential mechanisms for preventing cochlear damage that mouse models cannot replicate, with *Hras* emerging as the most significant hub upregulator. Functional validation in mice demonstrates that HC‐specific Hras overexpression significantly attenuates noise‐induced HC death, synaptic loss, and auditory threshold shifts. Mechanistically, Hras confers protection by activating the PI3K/Akt signaling pathway, a critical pro‐survival cascade. The findings further disentangle the mechanisms of cochlear resistance to intense noise in echolocating bats and suggest that targeting Hras expression may be a potential therapeutic intervention against NIHL.

## Graphical abstract

Comparative cochlear transcriptomics of noise‐exposed bats (*Miniopterus fuliginosus*) and mice reveals bat‐specific protection mechanisms for noise‐induced hearing loss (NIHL), identifying *Hras* as a key hub regulator. Functional studies in mice show hair‐cell‐specific Hras overexpression significantly reduced hair‐cell damage and NIHL by activating the pro‐survival PI3K/Akt pathway. Targeting Hras represents a potential NIHL therapy.

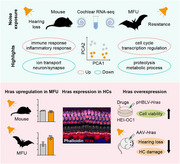

## 1 Introduction

In mammals, cochlear mechanosensory hair cells (HCs), including outer hair cells (OHCs) and inner hair cells (IHCs), play critical roles in hearing. Sound‐induced vibrations propagate along the cochlear basilar membrane, and OHCs amplify the mechanical signals while IHCs transduce the mechanical stimuli into electrical signals that are transmitted to the cochlear neurons through an elaborate presynaptic apparatus that relays the auditory input to the brain.^[^ ^1^, ^2^, ^3^ ^]^ Noise‐induced hearing loss (NIHL) refers to hearing loss caused by intense noise that leads to irreversible HC death and synaptopathy.^[^ ^4^ ^]^ Unlike HCs in lower vertebrates, mature HCs in the mammalian cochlea cannot spontaneously regenerate.^[^ ^5^ ^]^ Thus, identifying ways to protect HCs is critical for preventing NIHL. Traditionally, rodents such as mice, rats, and guinea pigs have been useful for modeling NIHL and studying experimental treatments, but these pathological models provide only a limited understanding of the natural mechanisms of NIHL resistance.

Echolocating bats rely extensively on their auditory systems to forage, navigate, and avoid obstacles. Previous studies have shown that echolocating bats exhibit natural resistance to NIHL and noise‐induced HC damage,^[^ ^6^, ^7^, ^8^, ^9^ ^]^ and this protective effect may be attributed to cellular and molecular adaptations to their high‐intensity echolocation calls.^[^ ^10^, ^11^ ^]^ Furthermore, the vocalization‐induced middle ear muscle reflex and auditory fovea have limited contribution to the unimpaired auditory sensitivity after noise exposure,^[^ ^12^ ^]^ strongly implicating a significant role of cochlear molecular heterogeneity in the intrinsic protective effects of echolocating bats. In our previous work, we compared the cochlear transcriptomes between echolocating bats and non‐echolocating fruit bats that do not experience intense‐noise stimulation and suggested some potential candidates that may contribute to their resistance to intense noise.^[^ ^9^ ^]^ However, there is still limited knowledge regarding the molecular mechanisms of natural resistance to intense‐noise stimulation, and the discovery of these mechanisms will bring new insights to our understanding of NIHL resistance.

In this study, we performed combined transcriptomic analyses of noise‐damaged cochleae in mice and noise‐resistant cochleae in the echolocating eastern bent‐winged bat (*Miniopterus fuliginosus*; MFU). After noise exposure, the mouse cochleae showed elevated immune and inflammatory responses and a decline in ion transport and synaptic function, while these changes were not seen in MFU cochleae. The specific transcriptional features in the MFU cochlea might therefore provide protection against NIHL. Of these, we experimentally confirmed that Hras overexpression in HCs protects against NIHL with decreased OHC death and IHC synaptopathy, and the underlying mechanism was shown to involve the activation of the PI3K/Akt pathway. Our findings thus suggest that Hras might be a new target for therapeutic interventions against NIHL.

## 2 Results

### 2.1 Differential Transcriptional Dynamics between the Mouse and MFU Cochlea After Noise Exposure

**Figure 1:**
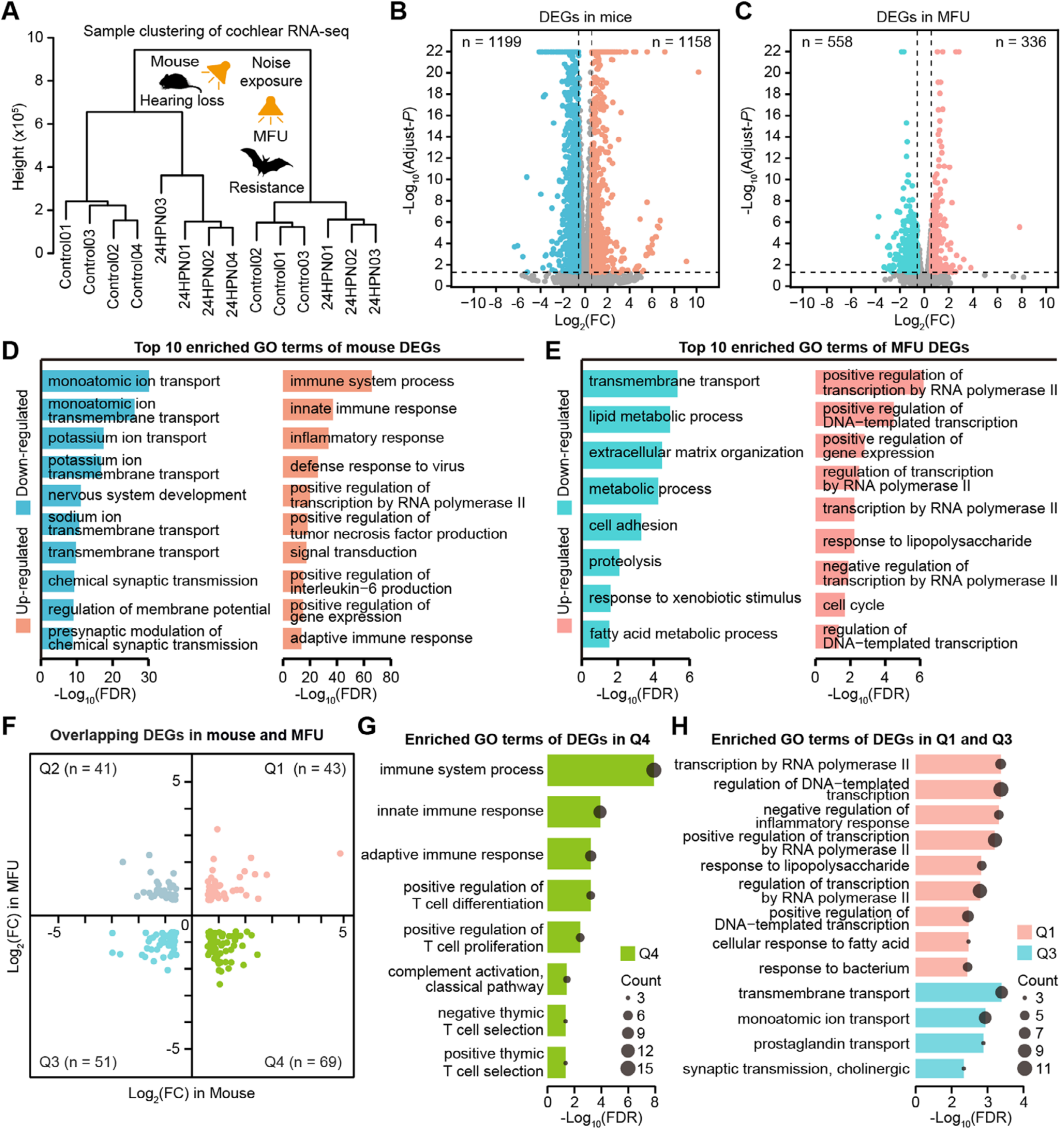
Differential transcriptional dynamics after noise exposure between the mouse and MFU cochlea. A) Clustering analysis of cochlear RNA‐seq samples from mouse and MFU cochleae. The samples of control and 24 h post noise exposure (24HPN) in the two species are labeled numerically. The data showed that the same cochlear sample models tended to cluster together. B,C) The volcano plot shows the differentially expressed genes (DEGs) in 24HPN versus control groups in the mouse cochlea (B) and MFU cochlea (C). The orange dots indicate up‐regulated genes, the blue dots indicate down‐regulated genes, and the grey dots indicate filtered genes. D,E) Top10 enriched GO terms of the mouse DEGs (D) and MFU DEGs (E). F) The overlapping DEGs in mice and MFU were divided into four groups: co‐up‐regulated DEGs in quadrant 1 (Q1), mouse down‐regulated and MFU up‐regulated DEGs in quadrant 2 (Q2), co‐down‐regulated DEGs in quadrant 3 (Q3), and mouse up‐regulated and MFU down‐regulated DEGs in quadrant 4 (Q4). G) The enriched GO terms of DEGs in Q4. H) The enriched GO terms of DEGs in Q1 and Q3. FC, fold change. GO, Gene Ontology. FDR, false discovery rate. The values for ‐log_10_(FDR) indicate the relative enrichment. *n* shows the numbers of genes for each group.

In our previous study, we reported that MFU maintains robust hearing sensitivity after noise exposure (4–24 kHz; 120 dB SPL for 2 h),^[^ ^9^ ^]^ while in mice the same stimulation results in severe loss of HCs along the entire cochlea at 24 h post noise exposure (24HPN), resulting in NIHL.^[^ ^13^ ^]^ This noise bandwidth and intensity are sufficient for the mice and MFU, given their similar hearing thresholds at 8, 16, and 24 kHz.^[^ ^9^ ^]^ We reasoned that cochlear transcriptome sequencing (RNA‐seq) would inform our understanding of the molecular mechanisms underlying the resistance to intense noise in MFU. Because MFU genomic data are unavailable for genome‐wide transcriptome analysis, in the present work, we sequenced the MFU genome using PacBio technology and generated ≈179 Gb long‐read sequences. The assembly was further polished with Illumina short reads and showed relatively high continuity (contig N50: ≈51.9 Mb). The final assembled MFU genome was ≈2 Gb in size. The benchmarking of universal single‐copy orthologs (BUSCO) analysis showed that we retrieved 95.7% of the complete BUSCO genes. Based on these findings, we conducted RNA‐seq of control and 24HPN cochleae using MFU as the noise‐resistant model. To ensure the reliability of the data, three biological replicates were performed for each group, yielding 35.14 Gb of high‐quality sequencing data across 6 samples (Table S1, Supporting Information). We also re‐analyzed our previously reported RNA‐seq data from control and 24HPN cochleae using mice as the noise‐damaged model (Table S1, Supporting Information).^[^ ^13^ ^]^ We obtained a total of 14318 one‐to‐one orthologous protein‐coding genes in mice and MFU using Inparanoid v4.1^[^ ^14^ ^]^ for the subsequent analyses. The sampletree clustering analysis showed that the samples from the same group tended to cluster together, indicating the strong repeatability and reliability of our transcriptomic data (**Figure** **1**A). We next performed the analysis of differentially expressed genes (DEGs) using the available expression data in mice and MFU, respectively. We identified 1199 down‐regulated DEGs and 1158 up‐regulated DEGs in the mouse cochlea (Figure 1B; Table S2, Supporting Information) and 558 down‐regulated DEGs and 336 up‐regulated DEGs in the MFU cochlea (Figure 1C; Table S3, Supporting Information). This result suggested that the noise‐resistant cochlea of MFU showed fewer molecular disturbances compared to the noise‐damaged cochlea of mice after noise exposure.

We next assessed the biological functions of the DEGs in mice and MFU according to the enrichment of Gene Ontology (GO) and Kyoto Encyclopedia of Genes and Genomes (KEGG) annotation terms. In the mouse cochlea, the top 10 enriched GO terms for the down‐regulated DEGs mainly involved ion transport and synaptic transmission, while the up‐regulated DEGs mainly involved immune and inflammatory responses (Figure 1D; Tables S4 and S5, Supporting Information). In the top 10 enriched KEGG terms for the down‐regulated DEGs mainly involved neuro/synapse activition (Figure S1A, Supporting Information), such as synaptic vesicle cycle (FDR = 7.88E‐06), neuroactive ligand‐receptor interaction (FDR = 2.56E‐06), glutamatergic synapse (FDR = 1.25E‐04), and GABAergic synapse (FDR = 0.00106), while the up‐regulated DEGs mainly implicated in immune and inflammatory processes, such as NF‐kappa B signaling pathway (FDR = 3.78E‐09), TNF signaling pathway (FDR = 1.33E‐07), and JAK‐STAT signaling pathway (FDR = 2.08E‐07) (Figure S1B, Supporting Information). Consistent with previous transcriptomic studies in mice, these results suggest functional degeneration and elevated immune/inflammatory responses in noise‐damaged cochleae.^[^ ^15^, ^16^, ^17^, ^18^ ^]^ However, these changes were only weakly observed in MFU cochleae, in which DEGs were mainly involved in metabolic processes, proteolysis, transcription regulation, and cell cycle regulation (Figure 1E; Tables S6 and S7, Supporting Information).

Furthermore, we found a total of 204 overlapping DEGs in the mouse and MFU cochleae (Figure 1F; Table S8, Supporting Information), including 43 co‐up‐regulated DEGs (quadrant 1, Q1), 41 reversed DEGs with mouse down‐regulation and MFU up‐regulation (quadrant 2, Q2), 51 co‐down‐regulated DEGs (quadrant 3, Q3), and 69 reversed DEGs with mouse up‐regulation and MFU down‐regulation (quadrant 4, Q4). GO enrichment analyses showed that the GO terms related to immune response and T cell regulation were significantly enriched in the Q4 DEGs (Figure 1G), but not in Q1, Q2, or Q3 DEGs (there were no enriched GO terms in Q2; Figure 1H; Table S9, Supporting Information).

Together, our findings indicated significant transcriptional heterogeneity in mouse noise‐damaged and MFU noise‐resistant cochleae, and suggested the inhibited or conservative immune and inflammatory responses in the echolocating bats. The specific transcriptional features in the MFU cochlea after noise exposure are thus likely to be involved in resistance to NIHL.

### 2.2 Specific Up‐Regulation of *Hras* in the MFU Cochlea Might Contribute to HC Resistance to Intense Noise

**Figure 2:**
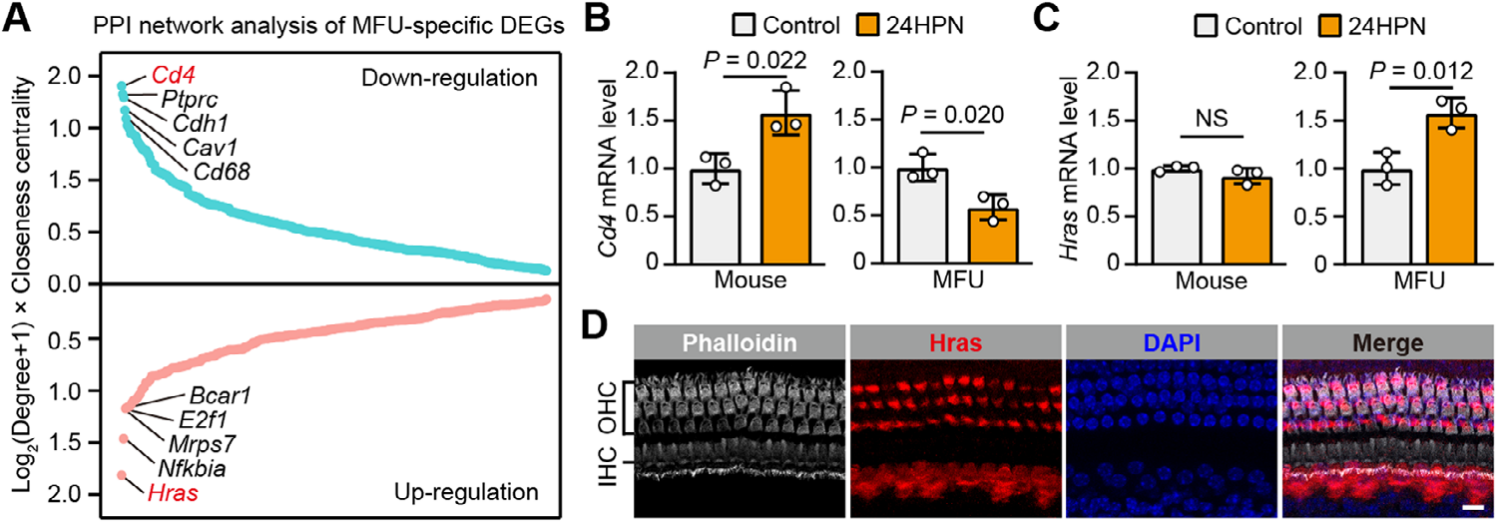
Hras is specifically up‐regulated in the MFU cochlea and shows self‐sustained expression in HCs. A) PPI network analysis of the DEGs with specific regulation in the MFU cochlea. The top 5 hub genes among the specific down‐ and up‐regulated DEGs are highlighted. *Cd4* and *Hras* were identified as the most down‐ and up‐regulated hub genes, respectively, among all the DEGs. B,C) The relative mRNA levels of *Cd4* (B) and *Hras* (C) in control and 24HPN mouse and MFU cochleae as determined by qRT‐PCR (n = 3 for each group). D) Representative confocal images showing the self‐sustained expression of Hras (red) in HCs (Phalloidin stain, white) from the cochlea of an intact adult mouse. Cell nuclei were labeled with DAPI (blue). Scale bar, 10 µm. All data are shown as the mean ± SD. NS, not significant. The *P*‐values are from two‐tailed Student's *t*‐tests.

We reasoned that the specific DEGs seen in the MFU cochlea would inform our understanding of the molecular basis of the natural resistance to intense noise seen in MFU. Therefore, we constructed a protein‐protein interaction (PPI) network using the STRING database for the specific DEGs in the MFU cochlea. We identified 587 nodes and calculated the product of log_2_(degree+1) and closeness‐centrality as the PPI score for each node in order to identify hub genes in the network (Table S10, Supporting Information). As shown in **Figure** **2**A, we identified *Cd4* as the top hub gene among the down‐regulated DEGs and *Hras* as the top hub gene among the up‐regulated DEGs. Furthermore, consistent with the transcriptome data, qRT‐PCR verified that *Cd4* decreased in the MFU cochlea and increased in the mouse cochlea after noise exposure, while *Hras* increased in the MFU cochlea but not in the mouse cochlea (Figure 2B,C).

NIHL largely results from injury to cochlear HCs, so we next investigated whether *Cd4* and *Hras* are expressed in HCs. However, a previous study reported that Cd4^+^ cells are rarely seen in the organ of Corti but are found in the modiolus, Rosenthal's canal, and spiral ligament.^[^ ^19^ ^]^ Notably, the expression profile of *Hras* is available from several HC transcriptome analyses.^[^ ^20^, ^21^, ^22^ ^]^ Consistent with these previous findings, our immunofluorescence results showed the self‐sustained expression of Hras in both OHCs and IHCs in adult mice (Figure 2D). Unfortunately, as a non‐model organism, the Hras status in the MFU cochlea could not be visualized because Hras primary antibodies showed limited effectiveness in both immunofluorescence and immunoblotting experiments (data not shown). Hras is a member of the Ras family of small GTPases, which play pivotal roles in cell proliferation, differentiation, and survival, as well as in immune responses and inflammation.^[^ ^23^ ^]^ Regardless, we suggest that Hras up‐regulation may play a key role in protecting HCs and preventing NIHL in echolocating bats.

### 2.3 Hras Overexpression Decreases Drug‐Induced Damage in HEI‐OC1 Cells

**Figure 3:**
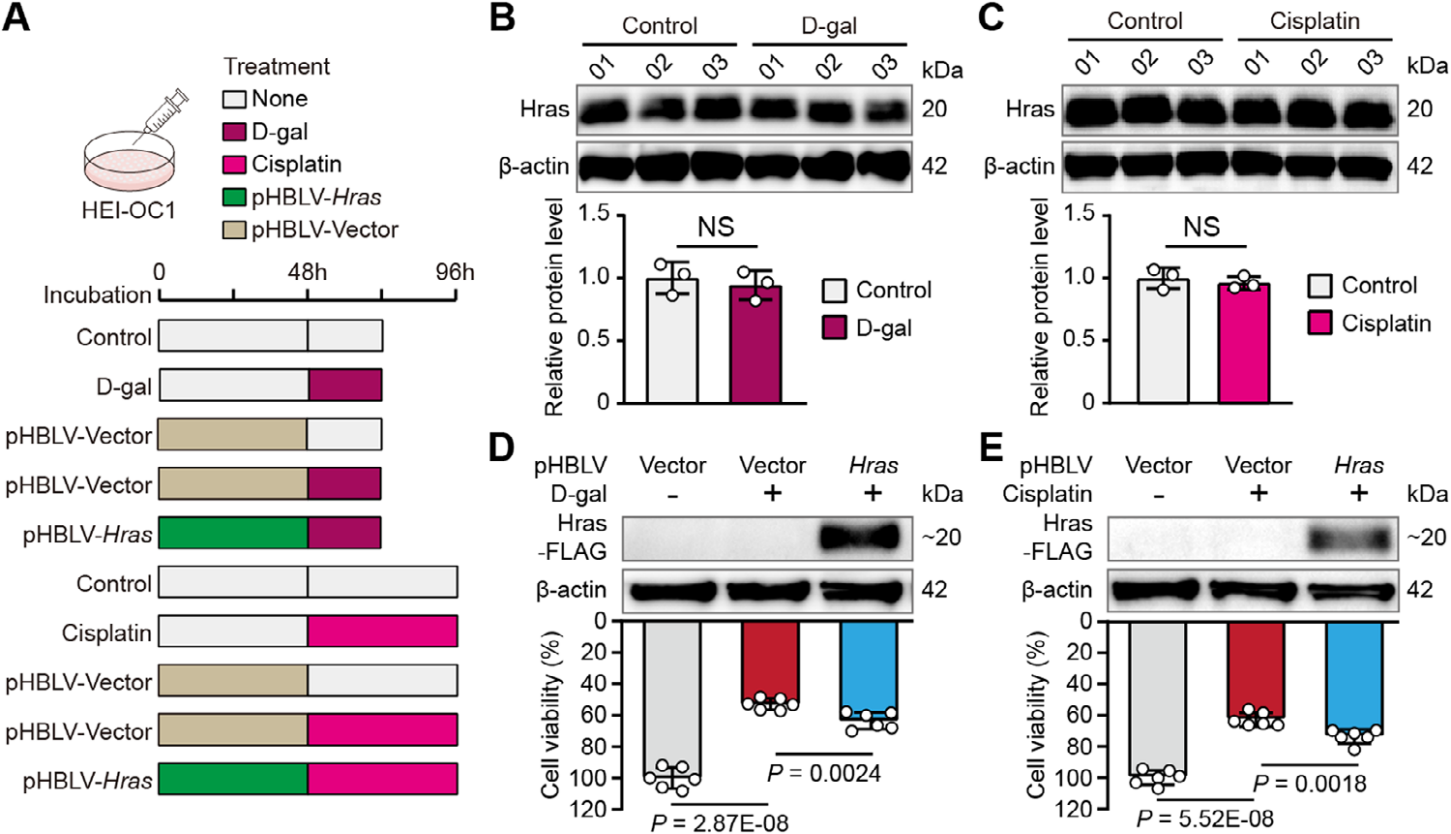
Hras overexpression decreases drug‐induced damage in HEI‐OC1 cells. A) The experimental treatment schedule for the in vitro experiments in HEI‐OC1 cells. The different treatments are indicated by different colors. B,C) Representative bands (upper panel) and quantitative analysis (lower panel) of immunoblotting showing the expression level of Hras in HEI‐OC1 cells after D‐gal (B) and cisplatin (C) treatments (n = 3 for each group). D,E) Representative immunoblotting bands of Hras‐FLAG (upper panel) and quantitative analysis of the cell viability (lower panel) for pHBLV‐Vector alone, pHBLV‐Vector+D‐gal, and pHBLV‐*Hras*+D‐gal treatment (D) in HEI‐OC1 cells and for pHBLV‐Vector alone, pHBLV‐Vector+cisplatin, and pHBLV‐*Hras*+cisplatin treatment (E) in HEI‐OC1 cells (n = 6 for each group). β‐actin was used as the loading control. All data are shown as the mean ± SD. NS, not significant. The *P*‐values are from two‐tailed Student's *t*‐tests.

House Ear Institute‐Organ of Corti 1 (HEI‐OC1) cells are widely used for investigating the molecular mechanisms behind the death and survival of cochlear HCs.^[^ ^24^ ^]^ To determine the protective effect of Hras up‐regulation, we induced cell damage using cisplatin (half‐lethal dose: 50 µm for 48 h) and D‐galactose (D‐gal; half‐lethal dose: 75 mg mL^−1^ for 24 h) treatments in HEI‐OC1 cells as in vitro models of HC degeneration, and we constructed the recombinant plasmid pHBLV‐*Hras*‐FLAG (pHBLV‐*Hras*) for Hras overexpression (**Figure** **3**A). In both models of drug‐induced HC degeneration, immunoblotting analyses showed no significant changes in Hras expression (Figure 3B,C), which was similar to what was seen for noise‐damaged cochleae in mice (Figure 2B), and the cell viability was significantly increased in the pHBLV‐*Hras* group compared to the pHBLV‐Vector group (Figure 3D,E). Notably, no cell viability changes were observed when Hras was overexpressed in HEI‐OC1 cells in the absence of drug‐induced damage (Figure S2, Supporting Information). These in vitro results indicated that Hras overexpression protects HEI‐OC1 cells against drug‐induced damage, strongly supporting the hypothesis that Hras up‐regulation contributes to HC resistance against intense noise.

### 2.4 Hras Overexpression Alleviates Noise‐Induced Auditory Brainstem Response (ABR) Threshold Shifts in Adult Mice

**Figure 4:**
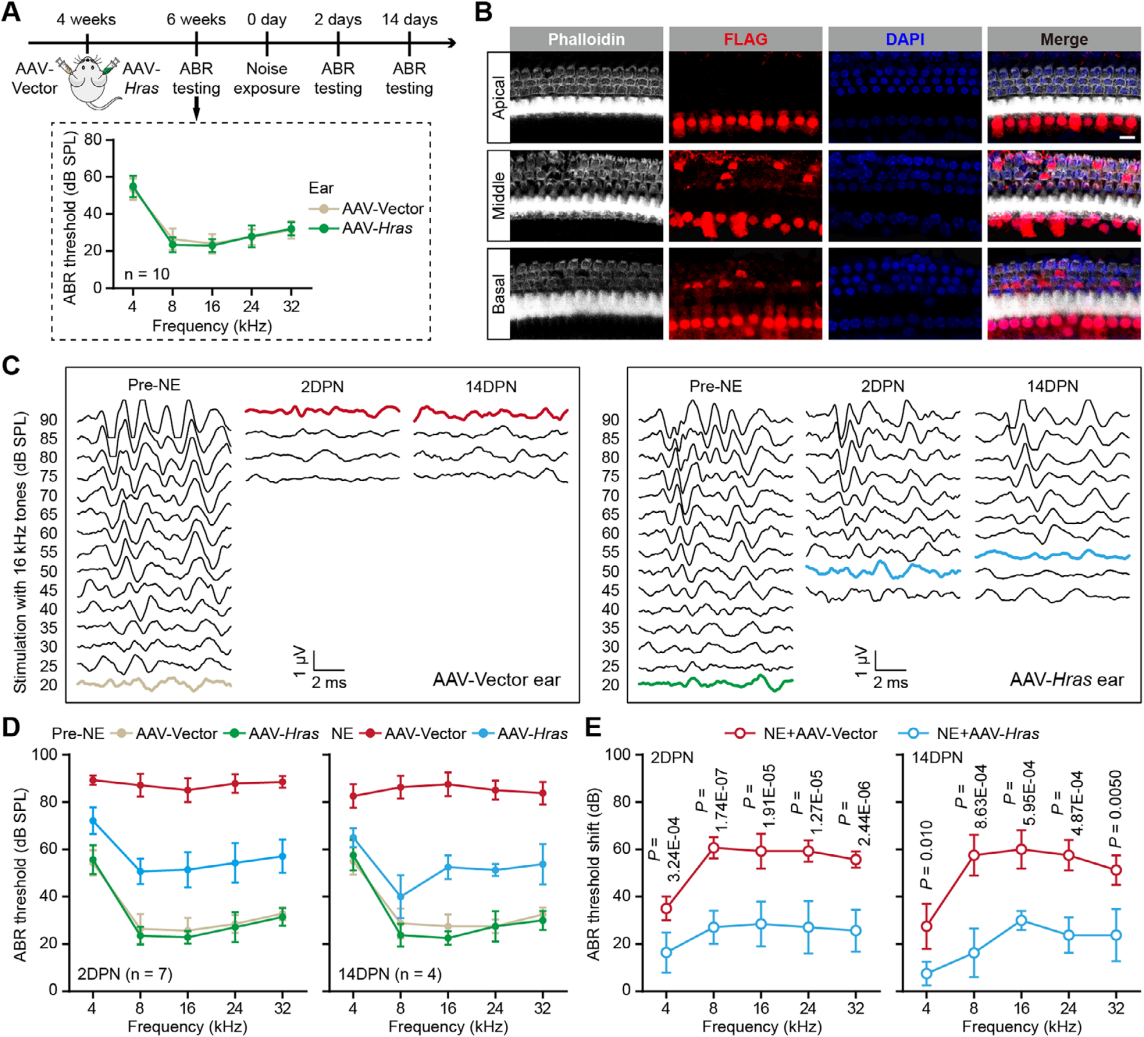
Hras overexpression alleviates noise‐induced ABR threshold shifts in adult mice. A) Experimental schedule (upper panel). Mice aged 4 weeks were injected with AAV‐*Hras* in one ear while the other ear of the same mouse was injected with the AAV‐Vector as the control, and noise exposure (NE) was performed 2 weeks later. Monaural ABR thresholds were measured at Pre‐NE, 2 days post NE (2DPN), and 14 days post NE (14DPN). The audiogram (lower panel) shows the monaural ABR thresholds of AAV‐Vector‐ and AAV‐*Hras*‐injected ears in response to different frequencies. B) Representative confocal images showing the extrinsic expression of Hras (FLAG stain; red) in HCs (Phalloidin stain; white) from the AAV‐*Hras*–injected cochlea. Cell nuclei were labeled with DAPI (blue). The cochleae were divided into the apical, middle, and basal turns. Scale bar, 10 µm. C) Representative monaural ABR waveforms of AAV‐Vector‐ and AAV‐*Hras*‐injected ears in response to 16 kHz tone bursts at Pre‐NE, 2DPN, and 14DPN. The ABR thresholds in each group are represented by colors. If there were no detectable ABR waveforms, the ABR thresholds were arbitrarily defined as 90 dB SPL for statistical analysis. D) Monaural ABR thresholds of AAV‐Vector‐ and AAV‐*Hras*‐injected ears in response to different frequencies at Pre‐NE, 2DPN, and 14 DPN. E) The ABR threshold shifts of AAV‐Vector‐ and AAV‐*Hras*‐injected ears at 2DPN and 14DPN compared to Pre‐NE. All data are shown as the mean ± SD. *n* shows the numbers of mice for each group. The *P*‐values are from two‐tailed Student's *t*‐tests.

The AAV‐PHP.eB vector has been reported to efficiently infect HCs,^[^ ^25^, ^26^ ^]^ so we constructed the recombinant AAV‐PHP.eB‐*Hras*‐FLAG virus (AAV‐*Hras*) to overexpress Hras in cochlear HCs in vivo. Because neonatal mouse HCs also have self‐sustained Hras expression (Figure S3, Supporting Information), we performed AAV delivery in 4‐week old C57BL/6 mice in order to avoid the potential impact of Hras overexpression on HC development. AAV‐*Hras* was delivered in one ear of the animal via the posterior semicircular canal approach, while the other ear was injected with the empty AAV‐Vector as the negative control, and ABR thresholds were measured 2 weeks later to assess whether the inner ear delivery of synthetic AAVs had any effect on hearing. Compared to the AAV‐Vector‐injected ears, the ears that underwent AAV‐*Hras* injections showed no significant changes in ABR thresholds at any test frequency (**Figure** **4**A). FLAG immunofluorescence showed robust extrinsic Hras expression in the IHCs along the entire AAV‐*Hras*‐injected cochlea and partial expression in OHCs in the middle and basal turns of the cochlea (Figure 4B). In addition, no distinct HC death or regeneration was observed when Hras was overexpressed.

Considering the limited Hras overexpression in OHCs in our experiment, we exposed mice to moderate noise levels (4–24 kHz; 115 dB SPL for 2 h) in the subsequent in vivo experiments in order to assess the potentially protective effects of Hras overexpression. As shown in Figure 4C–E, monaural ABR thresholds of the AAV‐Vector‐injected ears were significantly elevated at 2 days post noise exposure (2DPN) compared to pre‐noise exposure (Pre‐NE) measurements, which was persistent without any recovery observed at 14 days post noise exposure (14DPN). However, the ABR thresholds of the AAV‐*Hras*‐injected ears were less elevated at 2DPN compared to the AAV‐Vector‐injected ears, and this protective effect was also persistent at 14DPN. These results indicated that Hras overexpression in HCs protects against NIHL.

### 2.5 Hras Overexpression Protects HCs Against Noise‐Induced Damage in Adult Mice

**Figure 5:**
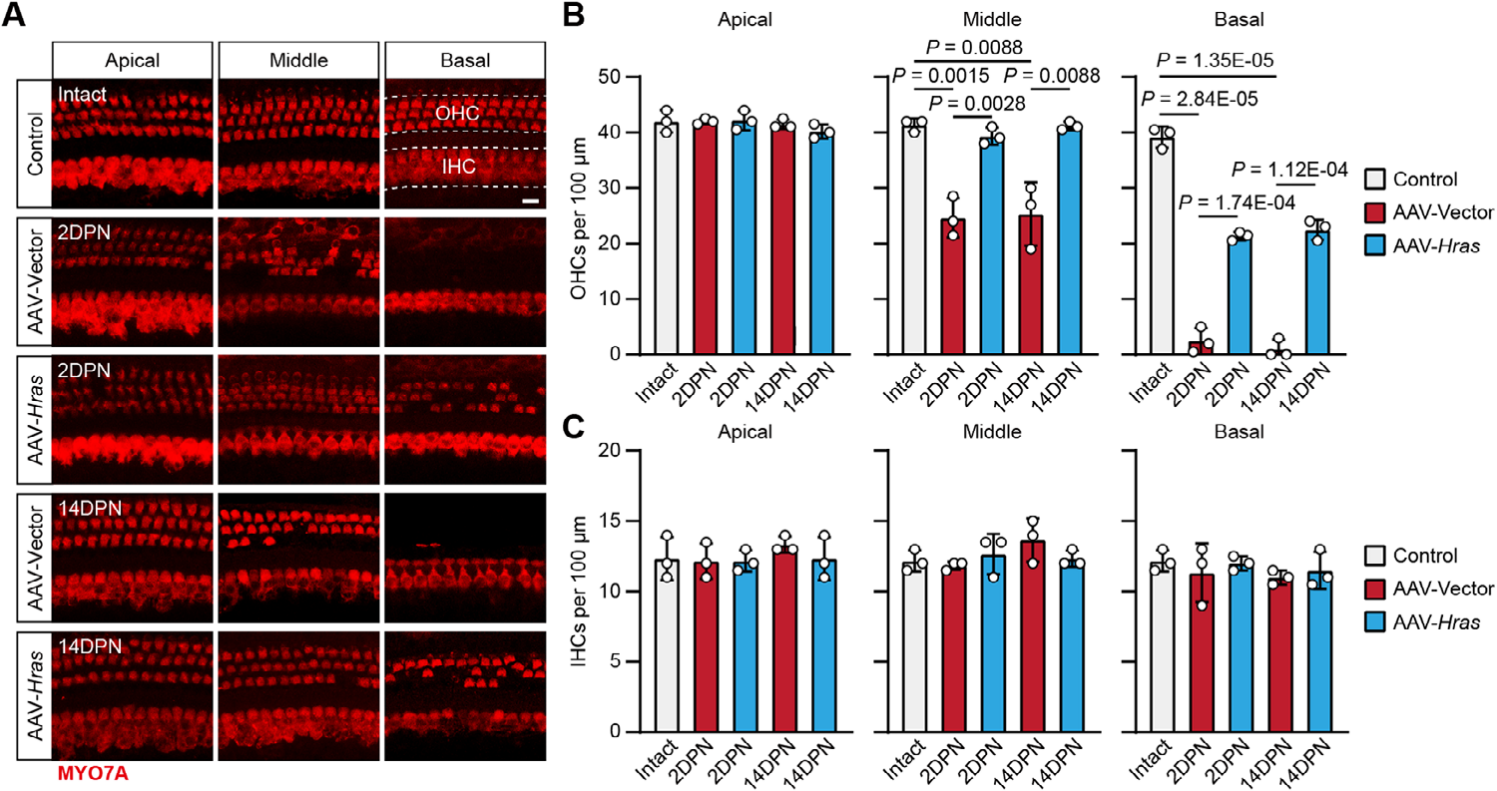
Hras overexpression protects OHCs against noise‐induced death in adult mice. A) Representative confocal images of cochlear HCs (MYO7A stain, red) from AAV‐Vector‐ and AAV‐*Hras*‐injected cochleae at 2DPN and 14DPN. The intact control cochleae were from adult C57BL/6 mice without any treatments. The cochleae were divided into the apical, middle, and basal turns. Scale bar, 10 µm. B,C) Quantification of the surviving OHCs (B) and IHCs (C) for each group. 2DPN, 2 days post noise exposure. 14DPN, 14 days post noise exposure. All data are shown as the mean ± SD, n = 3 for each group. The *P*‐values are from two‐tailed Student's *t*‐tests.

**Figure 6:**
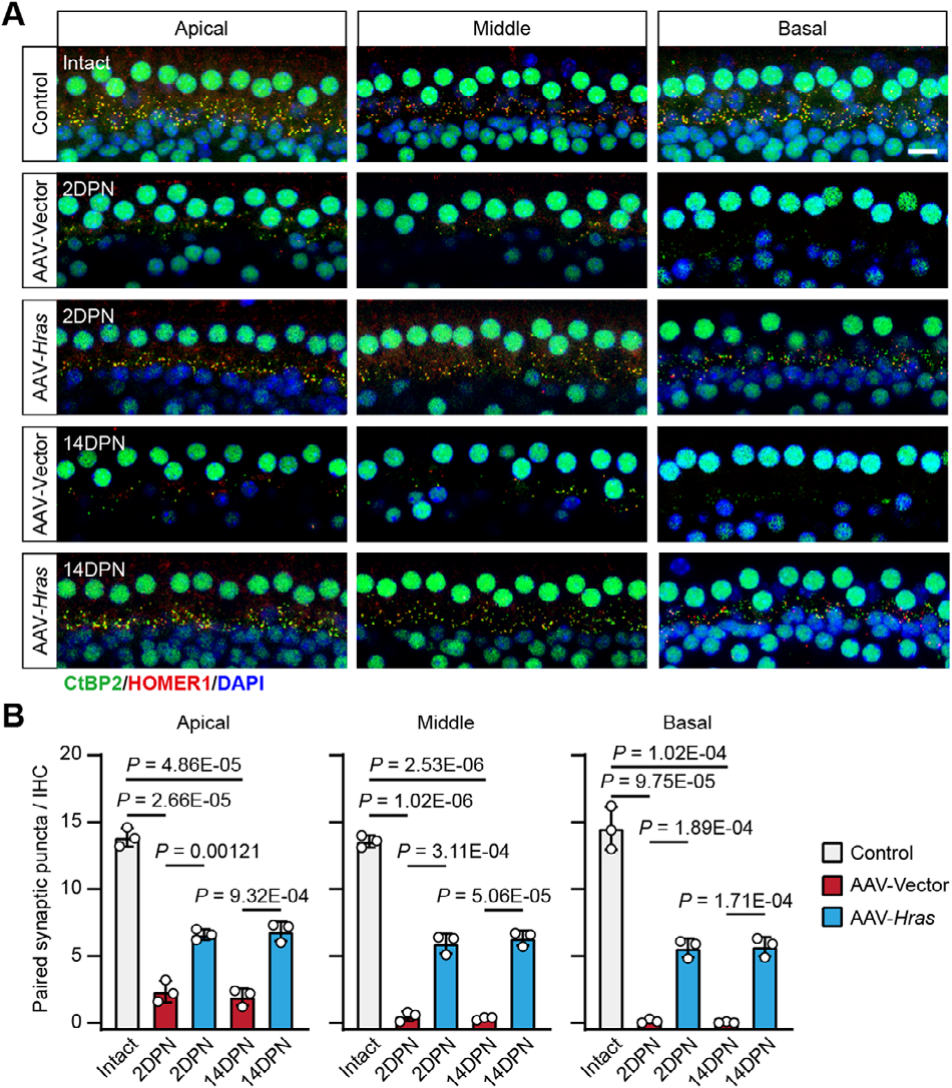
Hras overexpression protects IHCs against noise‐induced synaptopathy in adult mice. A) Representative confocal images of IHC ribbon synapses (presynaptic CtBP2 stain, green; postsynaptic HOMER1 stain, red) from AAV‐Vector‐ and AAV‐*Hras*‐injected cochleae at 2DPN and 14DPN. The nuclei were labeled with DAPI (blue). Postsynaptic HOMER1 puncta were weakly observed in the basal AAV‐Vector‐injected cochleae at both 2DPN and 14DPN in this case. The intact cochleae from adult C57BL/6 mice without any treatments were used as controls. The cochleae were divided into the apical, middle, and basal turns. Scale bar, 10 µm. B) Quantification of the remaining IHC synapses for each group. 2DPN, 2 days post noise exposure. 14DPN, 14 days post noise exposure. All data are shown as the mean ± SD, n = 3 for each group. The *P*‐values are from two‐tailed Student's *t*‐tests.

To assess the protective effect of Hras overexpression against NIHL at the morphological level, we first counted the number of cochlear HCs after noise exposure (**Figure** **5**A). Compared to the intact cochleae, the AAV‐Vector‐injected cochleae showed severe OHC loss in the middle and basal turns at both 2DPN and 14DPN; however, the OHC loss in AAV‐*Hras*‐injected cochleae was significantly attenuated compared to the AAV‐Vector‐injected cochleae (Figure 5B). In this case, there was no significant IHC loss in either AAV‐Vector‐ or the AAV‐*Hras*‐injected cochleae (Figure 5C). Because synaptopathy is another major pathology in noise‐exposed cochleae, we also counted the number of ribbon synapses in IHCs at 2DPN and 14DPN (**Figure** **6**A). As expected, at both 2DPN and 14DPN, the AAV‐Vector‐injected cochleae showed severe loss of IHC ribbon synapses in the entire cochlea, while ribbon synapse loss in AAV‐*Hras*‐injected cochleae was significantly attenuated compared to the AAV‐Vector‐injected cochleae (Figure 6B). Together, these results indicated that Hras overexpression attenuates HC damage resulting from acoustic trauma.

### 2.6 Hras Overexpression Activates the PI3K/Akt Pathway after Noise Exposure

**Figure 7:**
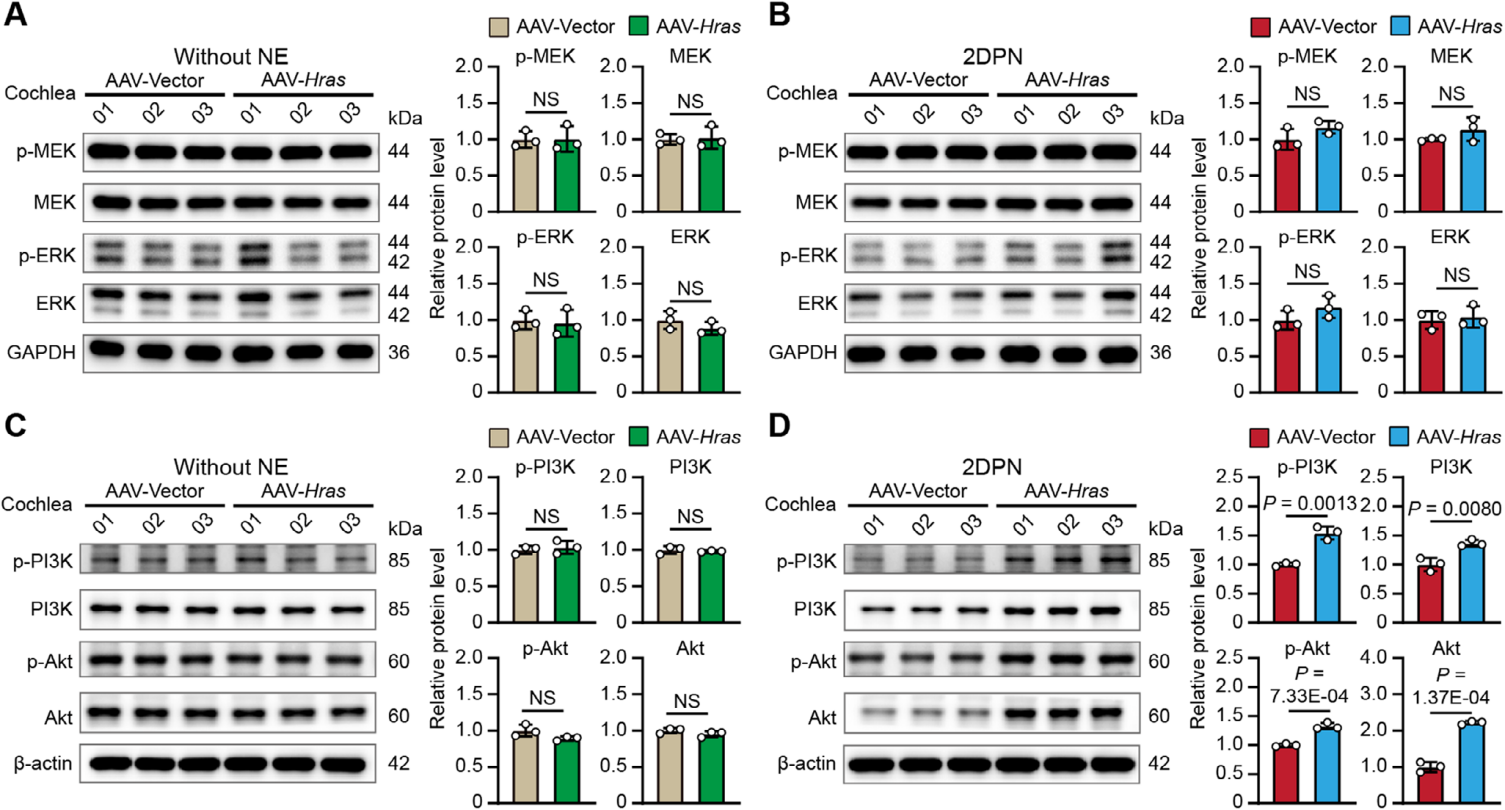
Hras overexpression activates the PI3K/Akt pathway. A,B) Representative bands and quantitative analysis of immunoblotting showing the expression level of p‐MEK, MEK, p‐ERK, and ERK in AAV‐Vector‐ and AAV‐*Hras*‐injected cochleae from mice without NE (A) and at 2DPN (B). C,D) Representative bands and quantitative analysis of immunoblotting showing the expression level of p‐PI3K, PI3K, p‐Akt, and Akt in AAV‐Vector‐ and AAV‐*Hras*‐injected cochleae from mice without NE (C) and at 2DPN (D). GAPDH and β‐actin were used as the loading controls. NE, noise exposure. 2DPN, 2 days post NE. All data are shown as the mean ± SD, n = 3 for each group. NS, not significant. The *P*‐values are from two‐tailed Student's *t*‐tests.

In response to extracellular stimulations, Ras signaling can mediate cell survival through two main signaling pathways, namely the Ras‐MAPK (MEK/ERK) pathway and the PI3K/Akt pathway.^[^ ^27^, ^28^ ^]^ Thus, we next performed immunoblotting assays to detect any changes in several critical factors of the MEK/ERK and PI3K/Akt pathways in the AAV‐Vector‐ and AAV‐*Hras*‐injected cochleae without noise exposure and at 2DPN (Figure S4, Supporting Information). In the MEK/ERK pathway, as shown in **Figure** **7**A,B, immunoblotting analyses showed no significant difference in the expression of p‐MEK, MEK, p‐ERK, or ERK between the AAV‐Vector‐ and AAV‐*Hras*‐injected cochleae without noise exposure or at 2DPN. In the PI3K/Akt pathway, the expression of p‐PI3K (Tyr458), PI3K, p‐Akt (Ser473), and Akt showed no significant differences between the AAV‐Vector‐ and AAV‐*Hras*‐injected cochleae without noise exposure (Figure 7C); however, these anti‐apoptotic proteins were significantly increased in the AAV‐*Hras*‐injected cochleae compared to the AAV‐Vector‐injected cochleae at 2DPN (Figure 7D). Together, these results indicated that Hras overexpression in HCs exerts a protective effect against NIHL at least partially by activating the PI3K/Akt pathway.

## 3 Discussion

Rodent models of NIHL have been extensively used for research into experimental treatments and have provided valuable insights into the pathogenesis of NIHL. In addition, echolocating bats exhibit a natural resistance to intense noise, offering an informative model for investigating anti‐NIHL mechanisms. In this study, we performed comparative analyses of cochlear transcriptomic differences after noise exposure between mice and MFU. Compared to mouse noise‐damaged cochleae, the transcriptional features of MFU noise‐resistant cochleae showed fewer molecular disturbances and reduced immune/inflammatory responses, strongly implying the existence of anti‐NIHL mechanisms in MFU. Thus, the specific regulators in the MFU cochlea suggest the presence of effective targets for NIHL prevention and therapeutics.

Given that *Hras* was identified as the top up‐regulated hub gene in MFU cochleae and showed self‐sustained expression in HCs, we performed functional and mechanical investigations into the protective effects of Hras overexpression on NIHL. In the mouse model, we found that Hras overexpression protected against noise‐induced HC damage and significantly attenuated ABR threshold elevation. Importantly, Hras overexpression did not disrupt baseline hearing, highlighting its therapeutic potential. Our mechanical studies indicated that the PI3K/Akt pathway was activated in Hras‐overexpressing cochleae after noise exposure. Consistent with this, studies have shown that blocking endogenous PI3K/Akt pathway activity increases the sensitivity to NIHL,^[^ ^29^ ^]^ and activation of the PI3K/Akt pathway induced by other treatments, such as activated protein C, PRMT5 inhibitors, and calpain inhibitors, can reduce HC damage and alleviate NIHL.^[^ ^30^, ^31^, ^32^ ^]^ Similar to our results, Hras was previously shown to protect against pressure overload‐induced cardiac dysfunction by promoting cardiomyocyte hypertrophy in part through activation of the PI3K/Akt pathway, and Hras was found to co‐immunoprecipitate with PI3K and to negatively regulate the expression of PTEN thus contributing to increased Akt activation.^[^ ^33^ ^]^ This may also be a possible explanation for the activation of the PI3K/Akt pathway when Hras is overexpressed in cochlear HCs.

Our in vitro experiments also indicated that Hras overexpression decreases D‐gal‐ and cisplatin‐induced damage in HEI‐OC1 cells (Figure 3D,E). D‐gal treatment mimics the alterations in cochlear HCs seen during aging,^[^ ^34^ ^]^ and cisplatin ototoxicity causes cochlear functional impairment and cellular degeneration,^[^ ^35^ ^]^ both resulting in irreversible hearing loss. Interestingly, as a major pro‐survival pathway, PI3K/Akt signaling also makes significant contributions to protecting against both cisplatin‐ and aminoglycoside‐induced ototoxicity.^[^ ^36^, ^37^, ^38^, ^39^ ^]^ In addition, immunofluorescence experiments in adult mice also showed self‐sustained expression of Hras in spiral ganglion neurons (SGNs) (Figure S5, Supporting Information). SGN insult is another indicator of cochlear both ototoxicity and age‐related damages,^[^ ^4^ ^]^ and this further supports the suggestion that Hras is a promising candidate for alleviating ototoxicity‐induced and age‐related hearing loss, although this should be verified in further prospective trials.

Although mutations in Ras family members make significant contributions to cancer, the activation of Ras signaling is tightly controlled in normal cells to regulate non‐oncogenic processes, thus highlighting its dual role in physiological and pathological conditions.^[^ ^40^, ^41^ ^]^ The normal functions of the Ras family are involved in cell proliferation, differentiation, migration, metabolism, apoptosis, and survival, and its proto‐oncogenic properties only manifest when the mutations result in sustained activation. Some of the pro‐survival mechanisms of Hras within different cells or tissues might provide additional possible processes to promote HC survival. For instance, Hras can promote hematopoietic cell survival by blocking the down‐modulation of Survivin (Birc5),^[^ ^42^ ^]^ and Birc5 expression has been found to be vital for protecting auditory cells against aminoglycoside‐induced cell death.^[^ ^43^ ^]^ Hras can inhibit the activity of apoptosis signal‐regulating kinase1 (ASK1) and thus can decrease ASK1‐induced apoptosis in the human embryonic kidney cell line,^[^ ^44^ ^]^ and ASK1 inhibition attenuates aminoglycoside‐induced HC death in vitro.^[^ ^45^ ^]^ In addition, Hras can attenuate IL‐1β‐induced chondrocyte apoptosis and extracellular matrix degradation in osteoarthritis,^[^ ^46^ ^]^ and IL‐1β has crucial roles in cochlear acoustic trauma, cisplatin ototoxicity, and aging.^[^ ^13^ ^]^ These findings further extend the relevance of Hras‐driven protection beyond NIHL, underscoring its versatility in hearing preservation research.

Beyond *Hras*, several additional hub genes that were up‐regulated in the MFU cochlea were found to be involved in particular kinds of hearing loss, including *Nfkbia*, *Mrps7*, and *Bcar1* (Figure 2A). Acoustic overstimulation generates reactive oxygen species accumulation that causes NF‐κB signaling to induce the overexpression of pro‐inflammatory cytokines in the cochlea,^[^ ^47^, ^48^ ^]^ and Nfkbia is a critical protein that inhibits NF‐κB nuclear translocation and impairs NF‐κB‐mediated signaling.^[^ ^49^, ^50^ ^]^ Mutation‐induced deficiency of Mrps7 causes a primary mitochondrial disorder leading to congenital hearing loss.^[^ ^51^ ^]^ Finally, Bcar1 prevents cisplatin‐induced hearing loss by promoting the repair of DNA damage in cochlear HCs.^[^ ^52^ ^]^ Thus, the up‐regulation of Nfkbia, Mrps7, and Bcar1 may also contribute to resistance to intense noise in echolocating bats.

In addition to the specific up‐regulated genes in the MFU cochlea, we also identified underlying protective mechanisms in specific down‐regulated genes, especially those in Q4, which suggested that the activation of immune cells, especially T cells, is inhibited in the MFU cochlea after noise exposure (Figure 1F,G). Some of these genes have been recognized as potential targets for ameliorating particular kinds of hearing loss, such as *Cd4*, *Cd45* (*Ptprc*), and *Cd68*, which we highlight in Figure 2A. Cochlear Cd4^+^ T cells are activated in cases of autoimmune hearing loss, and depletion of Cd4^+^ T cells prevents Lassa fever‐associated hearing loss in adult mice.^[^ ^53^, ^54^ ^]^ Cochlear Cd45^+^ immune cells, including natural killer cells, macrophages, and T cells, increase in number and infiltrate in response to acoustic trauma.^[^ ^17^, ^18^, ^55^ ^]^ Finally, Cd68 is a marker of macrophages, and reduced macrophage activation has been shown to protect against NIHL and cisplatin‐induced hearing loss.^[^ ^56^, ^57^ ^]^ Thus, the suppression of Cd4^+^, Cd45^+^, or Cd68^+^ immune cells may contribute to the resistance against intense noise in echolocating bats.

The hearing range of bats differs from that of mice. ABR recordings typically demonstrate sensitivity peaks not only within the high‐frequency range corresponding to their species‐specific echolocation calls but also within the lower frequency range crucial for perceiving social calls and sounds from predators and prey.^[^ ^58^ ^]^ Previous studies have shown that echolocating bats retain hearing sensitivity following exposure to broadband noise across various spectral ranges (e.g., 10–50 kHz, 10–80 kHz, 10–100 kHz, and 1–100 kHz).^[^ ^6^, ^7^, ^8^, ^12^ ^]^ These findings suggest an innate resistance to acoustic trauma throughout their auditory spectrum. In this study, the noise stimulus (4–24 kHz) was selected to target frequencies to which both MFU and mice are sensitive,^[^ ^9^ ^]^ providing a robust and comparable acoustic insult relevant to the auditory systems of both species. This approach effectively isolated intrinsic differences in their molecular responses to acoustic trauma, thereby revealing exceptional protective adaptations within the cochlea of echolocating bats.

Bats are the only mammals capable of self‐powered flight, and they possess some of the rarest mammalian adaptations, including laryngeal echolocation, exceptional longevity, and unique immunity.^[^ ^59^ ^]^ Hearing for echolocation behavior depends extensively on the cochlea, and similarly to their adaptations for their intense echolocation calls, laryngeal echolocating bats are resistant to cochlear aging and age‐related hearing loss.^[^ ^60^, ^61^ ^]^ In addition, echolocating bats are natural carriers for many pathogens because they show an excellent balance between enhanced host defense responses and immune tolerance or dampening.^[^ ^62^, ^63^ ^]^ Understanding these genetic adaptations may provide crucial insights into the prevention and treatment of hearing loss in humans, bridging evolutionary biology with translational medicine.

In conclusion, our study provides new insights into natural NIHL resistance in echolocating bats, and functional analyses confirmed that HC‐specific Hras overexpression protects against NIHL. These findings offer a promising foundation for developing novel NIHL therapies, with potential applications for age‐related and ototoxic hearing loss. Future research should focus on translating these mechanisms into clinical interventions, paving the way for innovative hearing protection strategies.

## 4 Experimental Section

### Ethical Regulations Statement

All animal care and experiments were performed under animal use protocols approved by the Kunming Institute of Zoology Animal Care and Ethics Committee, Chinese Academy of Sciences (IACUC‐PA‐2021‐06‐013).

### Plasmid and Virus Construction

For Hras overexpression, the coding sequence of the mouse *Hras* gene with a C‐terminal FLAG tag was cloned into the plasmid vector pHBLV and the AAV vector AAV‐PHP.eB, respectively. The coding sequence of the mouse *Hras* gene (CCDS22003.1) was from the CCDS database of the National Center for Biotechnology Information. The recombinant pHBLV‐Hras‐FLAG plasmid was packaged by Sangon Biotech (Shanghai, China), and the recombinant AAV‐PHP.eB‐Hras‐FLAG plasmid was packaged by PackGene Biotech (Guangzhou, China).

### Animal Surgery

The posterior semicircular canal (PSCC) approach was performed for inner ear gene delivery following established protocols.^[^ ^64^ ^]^ In brief, a polyimide tube of 0.1 mm diameter was connected to a glass micropipette (504949; WPI, Sarasota, FL, USA) and attached to a Nanoliter Microinjection System (NANOLITER2020; WPI Sarasota, FL, USA). C57BL/6 mice aged 4 weeks were anesthetized with an intraperitoneal injection of sodium pentobarbital (100 mg kg^−1^). The anesthetized mice were placed on a 42 °C heating pad during the surgical procedure and recovery. The surgery was performed under an operating microscope. The post‐auricular region was shaved and cleaned, and a post‐auricular incision was made using small scissors. The sternocleidomastoid muscle was divided to expose the PSCC, and PSCC fenestration was made with a microprobe. The leakage of lymph confirmed successful access to the PSCC lumen. After the efflux abated, the tip of the polyimide tube was inserted into the hole through the fenestration site and then sealed with tissue adhesive (3 M Vetbond). A total of 2 µL AAV (≈4 × 10^10^ GCs) was injected into the cochlea through the polymide tube at a rate of 5 nL s^−1^. After injection, the tubing was removed, and the residual hole was sealed with tissue adhesive. The incision was then closed with sutures.

### Noise Exposure

Animals were exposed to broadband noise (4–24 kHz) at 120 dB SPL or 115 dB SPL for 2 h in a small cylindrical cage as previously described.^[^ ^9^, ^13^ ^]^ The noise was delivered by a loudspeaker, and the calibration of noise to target SPL was performed immediately before each noise exposure session to ensure that the SPL varied by < 1 dB across the cage.

### ABR Measurement

The animals were anesthetized by intraperitoneal injection of sodium pentobarbital (100 mg kg^−1^), and the animal's body temperature was maintained at 37 °C by a heating pad during the recordings. A subdermal recording electrode was located over the skull vertex, and the reference electrode was placed ventrolaterally to the external pinna. Each ear was individually stimulated using the tube of a close‐field speaker (MF1; Tucker‐Davis Technology, Alachua, FL, USA), and all measurement signals were recorded using BioSigRZ software and RZ6 hardware (Tucker‐Davis Technologies, Alachua, FL, USA). The loudspeaker was placed ≈2 cm in front of the animal's nose, and tone bursts (4, 8, 16, 24, and 32 kHz) were delivered at intensities of 5–90 dB SPL in 5 dB intervals. The ABR signals were amplified, filtered (100–1000 Hz), and averaged (256 times) prior to storage for offline analysis. The ABR threshold was the lowest sound intensity at which reproducible waveforms could be visually distinguished. If there were no detectable ABR waveforms, the ABR thresholds were arbitrarily defined as 90 dB SPL for statistical analysis.

### Cochlear Fixation and Decalcification

To obtain the cochleae, the animals were anaesthetized by isoflurane inhalation and then rapidly decapitated. The cochleae were dissected in PBS, and a small hole was made in the apical region. The collected cochleae were fixed in 4% paraformaldehyde (PFA) overnight and then decalcified in 10% ethylene diamine tetraacetic acid (EDTA) solution for several days at room temperature (RT). After decalcification, the cochleae were transferred into PBS for temporary storage at 4 °C.

### Cochlear Tissue Fabrication

The decalcified cochleae were treated in two different ways. In the first method, the basilar membrane was divided into pieces under a stereoscopic microscope (SMA 745T; Nikon, Japan). In the second method, the decalcified cochleae were dehydrated with 20% sucrose solution followed by 30% sucrose solution, embedded in optimal cutting temperature (OCT) compound (4583; Sakura Finetek, USA), snap‐frozen in Drikold, and cut into 20 µm sections using a manual cryostat (CM3050 S; Leica, Germany).

### Immunostaining and Confocal Imaging

The cochlear tissues were permeabilized and blocked in a solution of 1% Triton‐X100 and 10% goat serum in PBS for 1 h at RT. Depending on the specific experimental requirements, the tissues were then incubated at 4 °C overnight with the primary antibodies (1:500–1:1000 dilution), including rabbit anti‐Hras (PA5‐88424; Thermo Fisher Scientific, USA), rabbit anti‐MYO7A (25‐6790; Proteus BioSciences, USA), rabbit anti‐HOMER1 (12433‐1‐AP; Proteintech, USA), mouse anti‐CtBP2 (AB‐399431; BD BioSciences, USA), mouse anti‐FLAG (MAB3118; Sigma–Aldrich, USA), and mouse anti‐Tuj1 (801202; BioLegend, USA). When necessary, the filamentous actin of HCs was labeled by a 30 min incubation with phalloidin conjugated to Alexa Fluor 647 (A22287; Thermo Fisher Scientific, USA). After three rinses with PBS, the tissues were incubated with the appropriate secondary antibodies and stained with DAPI to mark the cell nuclei. After three rinses with PBS, the tissues were mounted in an antifading mounting medium (S2100; Solarbio, China). With maximum intensity projections of z‐stacks, images were obtained on a confocal microscope platform (STELLARIS5; Leica, Germany) using a 10×, 20×, or 40× lens. For the observation of HCs, the numbers of MYO7A‐positive cells along 200 µm in each segment of the cochlea were counted. For the observation of synaptic ribbons, the CtBP2‐HOMER1‐positive puncta along at least 20 IHCs in each segment were counted.

### Cochlear Total RNA Collection

According to the experimental design, an individual animal's bilateral cochleae were collected as biological replicates. After removing the vestibular organ in a Petri dish filled with ice‐cold sample protector for RNA/DNA (9750; TaKaRa, Japan), the total RNA was extracted from the remaining cochleae using QIAzol lysis reagent (79306; Qiagen Science, Germany). Next, the total RNA was purified with an RNeasy Plus Universal Mini Kit (73404; Qiagen Science, Germany), and the quantity and integrity of the purified total RNA were examined using an Agilent 2100 Bioanalyzer (Thermo Fisher Scientific, USA). To ensure the reliability and repeatability of the data, at least three replicates were required.

### Quantitative Real‐Time PCR (qRT‐PCR)

For qRT‐PCR, the bilateral cochleae of each individual animal were collected for total RNA extraction as a biological replicate. The extracted total RNA was reverse transcribed into cDNA using a PrimeScript RT reagent kit with gDNA Eraser (RR047A; Takara, Japan). The qRT‐PCR assays were performed with GoTaq qPCR Master Mix (A6001; Promega, USA) using a Quant‐studio 12K Flex (AB Life Technologies, USA). The qRT‐PCR data were analyzed using the 2^–ΔΔCt^ method with *Gapdh* as the endogenous reference control.

The primer sequences used in this study were as follows: *Hras*: Forward 5′‐CAT CAA CAA CAC CAA GTC‐3′, Reverse 5′‐GGC ACA TCA TCT GAA TCT‐3′; *Cd4*: Forward 5′‐GGA AGA CTC TCA GAC TTA TAT C‐3′, Reverse 5′‐TGA AGG TCA CTT TGA ACA C‐3′; *Gapdh*: Forward 5′‐ACC ACC ATG GAG AAG GCC‐3′, Reverse 5′‐ATT GCT GAC AAT CTT GAG TGA GT‐3′. The primers were generated by Sangon Biotech (Shanghai, China).

### Genome Sequencing and Assembly

Multiple fresh tissues were obtained from a male MFU to sequence its genome with high accuracy. The genomic DNA was extracted from the muscle tissues, and its concentration, integrity, and purity were assessed using a Qubit 3.0 fluorometer (Invitrogen, USA). The long DNA fragments were obtained using a BluePippin system (Sage Science, USA). The sequencing of the library fragments was then performed on a Nanopore GridION X5/PromethION platform (Oxford Nanopore Technologies, UK). The total amount of clean data was 179 Gb, with 8199162 reads with an average length of 21.8 Kb, an N50 of 31.4 Kb, and a longest read length of 297 Kb. The NextDenovo software (v1.0) was used to correct the original data to ensure the consistency of the sequences using the following parameters: read_cutoff = 1k; seed_cutoff = 20k; blocksize = 2 g. The wtdbg software (v1.2.8) was then used to assemble the results using the following parameters: wtDBg‐1.2.8‐k 0 ‐p 17‐S 2 –rescue‐low‐cov‐edges –tide‐reads 10000; wtdbg‐cns ‐c 3 –k 13. The assembled genome size was 1928 Mb, the Contig N50 was 51.9 Mb, and the Contig number was 648. The assessment of the completeness of the assembled genome was based on the BUSCO method,^[^ ^65^ ^]^ resulting in 3927 (95.7%) of the 4104 single‐copy orthologous genes being used to assemble the MFU genome in the OrthoDB database mammalia_odb10.

### Annotation of Repetitive Elements in the MFU Genome

RepeatModeler (v1.0.4) was used to obtain a de novo repeat sequence library from the MFU genome.^[^ ^66^ ^]^ This library was combined with the mammalian repeat library extracted from the RepBase library to obtain a comprehensive repeat library.^[^ ^67^ ^]^ Using the comprehensive repeat library as the database, RepeatMasker was used to retrieve the repetitive elements in the MFU genome.^[^ ^68^ ^]^ Finally, 33.93% of the genome was masked as repetitive elements.

### Annotation of the MFU Genome

The Broad Institute eukaryotic annotation pipeline was used to predict protein‐coding genes in the MFU genome, including RNA‐seq‐based gene prediction, homology‐based gene prediction, and ab initio gene prediction.^[^ ^69^ ^]^ Finally, a fixed method was used to integrate the results of the above three parts. For the RNA‐seq–based gene prediction, transcriptomic data were first collected from four distinct MFU tissues (heart, liver, kidney, and brain). Trimmomatic (v0.39) was used with the default parameters to filter the raw RNA‐seq reads.^[^ ^70^ ^]^ To obtain a consensus gene set based on overlapping assembled transcripts, PASA was used to incorporate the different transcriptome assemblies generated using HISAT/StringTie and Trinity.^[^ ^71^, ^72^, ^73^, ^74^ ^]^ For homology‐based gene prediction, eight high‐quality genomes were collected, including human (*Homo sapiens*), mouse (*Mus musculus*), brown rat (*Rattus norvegicus*), dog (*Canis familiaris*), cow (*Bos taurus*), flying fox (*Pteropus vampyrus*), little brown bat (*Myotis lucifugus*), and the Natal long‐fingered bat (*Miniopterus natalensis*). TblastN was used to align all protein sequences from each species to the assembled genome, and GeMoMa was used to predict the gene structures.^[^ ^75^ ^]^ For ab initio gene prediction, the de novo prediction of gene models was performed using GlimmerHMM, geneid, genscan, and AUGUSTUS.^[^ ^76^, ^77^, ^78^, ^79^ ^]^ The above three gene prediction datasets were integrated using EVM to obtain a comprehensive and nonredundant gene set,^[^ ^69^ ^]^ and PASA was used to further update the EVM consensus predictions by adding untranslated region annotations and gene models for alternatively spliced isoforms.

To obtain a high‐quality set of protein‐coding genes, only those that met the following criteria were retrieved: 1) the length of the encoded amino acid sequences was ≥50; 2) the encoded protein sequences could be mapped to the National Center for Biotechnology Information (NCBI) nonredundant protein database with a cutoff of *E* < 10^−5^; 3) the ratio of the alignment length to the query length was ≥0.5; and 4) the ratio of the alignment length to the subject length was ≥0.5. Finally, a total of 23533 protein‐coding genes were obtained, which were highly comparable to results from other mammals with high‐quality genomic data.

### Cochlear Transcriptome Sequencing and Analysis

Approximately 1 µg of total RNA was used to construct cDNA libraries according to the manufacturer's recommendations. All the libraries were sequenced on the MGISEQ2000 platform (BGI‐Shenzhen, China) in a paired‐end form with 150 bp. The low‐quality reads were filtered out using the fastq‐quality‐filter from the Fastx‐Toolkit 0.0.13 with the following parameters: ‐Q 33 ‐q 20 ‐p 80. A total of 35.14 Gb of clean data was obtained with an average of 5.86 Gb high‐quality clean reads for MFU cochleae from the control and 24HPN groups. The raw data of control and 24HPN groups in mouse cochleae were downloaded from the Gene Expression Omnibus in the National Center for Biotechnology Information (GSE196870).

The clean reads were mapped onto the *Miniopterus fuliginosus* or *Mus musculus* (GRCm39) genomes using Hisat2‐2.1.0 with the parameter of –read‐mismatches 2. The expected counts of each gene were calculated using Stringtie‐2.1.6, and the Deseq2 R package was used for the analysis of the DEGs within the count data. The genes with an adjusted *P*‐value < 0.05 and fold‐change ≥ 1.5 were considered DEGs.

### Functional and Pathway Enrichment Analysis and PPI Network Construction

The GO and KEGG annotations were downloaded from DAVID Bioinformatics Resources (david.ncifcrf.gov) and were used to assign functional categories to one‐to‐one orthologous genes. Using all of the mouse genes as the background, an in‐house Fisher's exact test program was used to perform the statistical analysis. The PPI network was constructed using STRING (string‐db.org/cgi/input). The number of degrees and the closeness‐centrality value for each gene involved in the networks were calculated with Cytoscape 3.8.0.^[^ ^80^ ^]^

### In vitro Experiments in HEI‐OC1 Cells

Cell culture, plasmid cDNA transfection, and cisplatin and D‐gal treatment in HEI‐OC1 cells were performed following the previous work.^[^ ^13^ ^]^ In brief, the HEI‐OC1 cells were cultured in Dulbecco's Modified Eagle's Medium (Gibco, USA) containing 10% fetal bovine serum (Gibco, USA) without antibiotics at 33 °C and 5% CO_2_. Cells were seeded in a 96‐well plate at a density of 1.0 × 10^4^ cells per well or in a 6‐well plate at a density of 1.0 × 10^5^ cells per well and incubated overnight. Next, the plasmid was transfected using Lipofectamine 3000 reagent (L300015; Invitrogen, USA) and Opti‐MEM (31985062; Gibco, USA). The HEI‐OC1 cells in the 96‐well plates were transfected with 0.2 µg plasmid in 0.3 µL Lipofectamine 3000, 0.2 µL P3000, 10 µL Opti‐MEM, and 100 µL complete growth medium, and the HEI‐OC1 cells in the 6‐well plates were transfected with 5 µg plasmid in 7.5 µL Lipofectamine 3000, 5 µL P3000, 250 µL Opti‐MEM, and 2 mL complete growth medium. The respective empty plasmid vectors were transfected as controls. After 48 h of transfection, the compound medium was removed and replaced by complete growth medium with 50 µm cisplatin (P4394; Sigma–Aldrich, USA) for a 48 h incubation or with 75 mg mL^−1^ D‐gal (CG5841; Coolaber, China) for a 24 h incubation.

### Assessment of Cell Viability

The assessment of cell viability in 96‐well plates was performed using a Celltiter 96 AQueous One Solution Cell Proliferation Assay (G3581; Promega, USA). After treatment with D‐gal or cisplatin, 10 µl compound was added for 2 h, and the optical density (OD) values were measured at 450 nm using a Hybrid reader (Synergy H1; BioTek, USA). The positive controls underwent the same procedure but without cell seeding. The average OD in the negative control cells was considered 100% viability. The relative viability was calculated as (OD experiment – OD positive)/(OD negative – OD positive) × 100. The negative control was HEI‐OC1 cells without any treatment.

### Immunoblotting Analysis

The HEI‐OC1 cells from the 6‐well plates were collected, and total protein was extracted using ice‐cold radioimmunoprecipitation assay (RIPA) lysis buffer for 30 min. The unilateral cochleae were collected in ice‐cold RIPA lysis buffer for 1 h and then crushed using grinding beads in a low‐temperature automatic tissue grinder (LUKYM‐II; Guangzhou, China). After centrifugation at 12000 × *g* at 4 °C for 15 min, the protein concentration was determined using the BCA assay (P0009; Beyotime Biotech, China). Subsequently, equal amounts of protein samples underwent separation via 10% sodium dodecyl sulfate‐polyacrylamide gel electrophoresis followed by transfer to a polyvinylidene fluoride membrane (Millipore, Bedford, MA, USA). After blocking with 5% non‐fat milk in TBST for 2 h at RT, the blots were incubated with the primary antibodies (1:500–1:10 000 dilution) overnight at 4 °C, including rabbit anti‐Hras (PA5‐88424; Thermo Fisher, USA), rabbit anti‐PI3K (4292; Cell Signaling Technology, USA), rabbit anti‐p‐PI3K (17366; Cell Signaling Technology, USA), rabbit anti‐AKT (9272; Cell Signaling Technology, USA), rabbit anti‐p‐AKT (4060; Cell Signaling Technology, USA), rabbit anti‐MEK1/2 (ab178876; Abcam, UK), rabbit anti‐p‐MEK1/2 (9154; Cell Signaling Technology, USA), rabbit anti‐ERK1/2 (9102; Cell Signaling Technology, USA), rabbit anti‐p‐ERK1/2 (4370; Cell Signaling Technology, USA), mouse anti‐FLAG (MAB3118; Sigma–Aldrich, USA), mouse anti‐β‐actin (A1978; Sigma–Aldrich, USA), and mouse anti‐GAPDH (AF0006; Beyotime Biotech, China). Following a TBST wash, the polyvinylidene fluoride membrane was incubated with HRP‐conjugated secondary antibodies (SA00001‐1 and SA00001‐2, Proteintech, USA) at RT for 2 h. Finally, the chemiluminescence (ECL) detection reagents (P0018S; Beyotime Biotech, China) were used to detect the target protein signals, and the band images were captured using a Tanon 5200 imaging system (Tanon Science & Technology, China). Band intensities were quantified using ImageJ, and the relative density ratio was calculated by comparison to β‐actin or GAPDH. The original immunoblot bands are shown in Figure S6 (Supporting Information).

### Statistical Analysis

The experimental data were presented as Mean ± SD. Statistical comparisons between two groups were performed using an unpaired Student's *t*‐test, derived from a minimum of three independent experiments. A significance threshold of *P* < 0.05 was applied for all tests. All experimental data analysis and statistical evaluation were performed using GraphPad Prism 9 software.

## Conflict of Interest

The authors declare no conflict of interest.

## Supporting information



Supporting Information

Supporting Information

## Data Availability

The RNA‐seq sequencing data generated in this study was deposited at the database of the National Center for Biotechnology Information (PRJNA1229455). This Whole Genome project has been deposited at GenBank under the accession JBLYEM000000000 (PRJNA1230585). The version described in this paper is version JBLYEM010000000. These sequencing data generated in this study have also been deposited in the Science Data Bank [https://doi.org/10.57760/sciencedb.21572].
