# Supplementary material for: Comparative Cochlear Transcriptomics in Echolocating Bats and Mouse Reveals Hras as Protector Against Noise‐Induced Hearing Loss
Source: Adv Sci (Weinh). 2025 Sep 8;12(44):e08466. doi: 10.1002/advs.202508466 (PMC12667500; doi:10.1002/advs.202508466)
Supplement: Supplementary file 1 — Supporting Information [file ADVS-12-e08466-s002.doc]

**
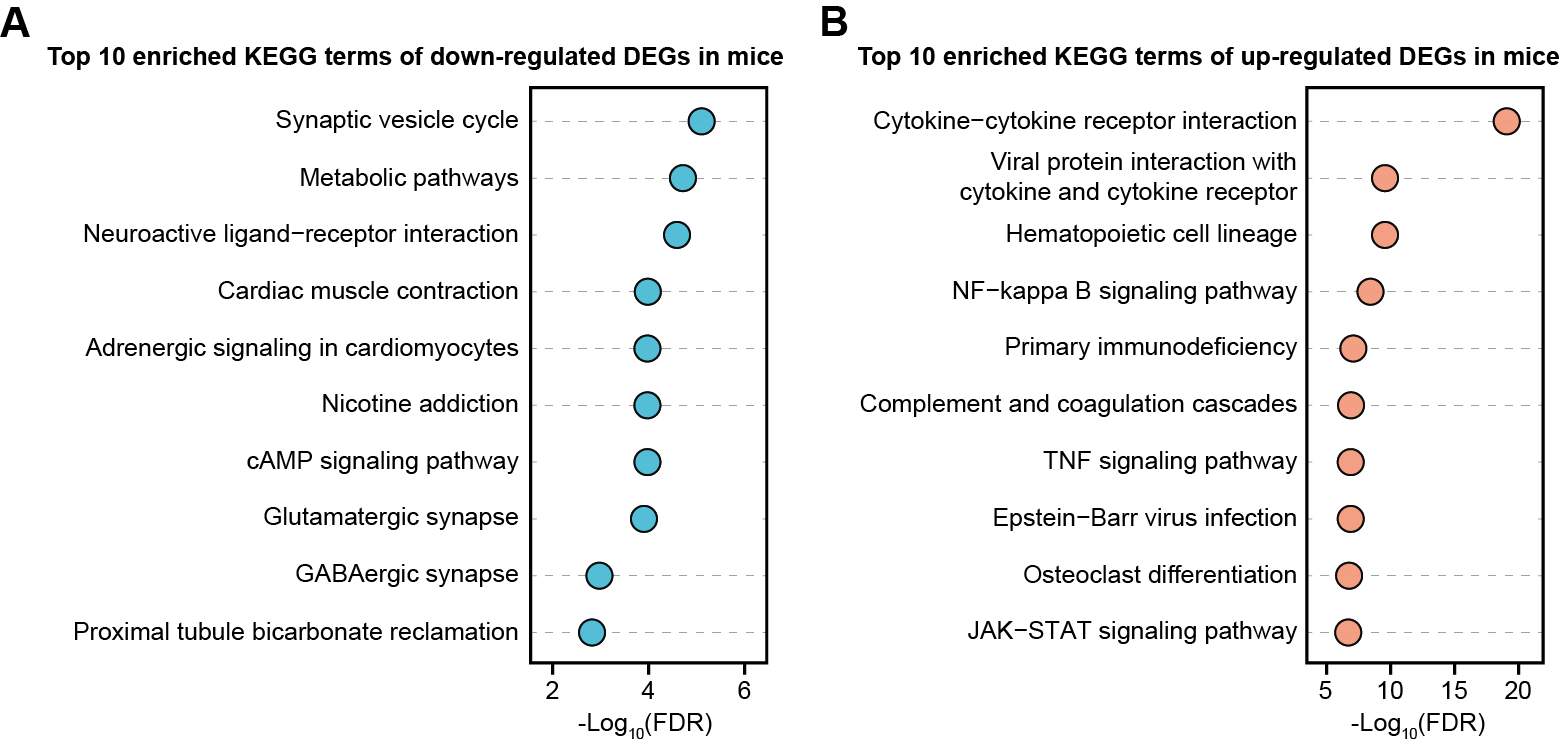
**

**Figure S1.** KEGG enrichment analysis for the differentially expressed genes (DEGs) in mice. A and B) Top10 enriched KEGG terms of the mouse down-regulated DEGs (A) and up-regulated DEGs (B). KEGG: Kyoto Encyclopedia of Genes and Genomes. FDR, false discovery rate. The values for -log_10_(FDR) indicate the relative enrichment.

**
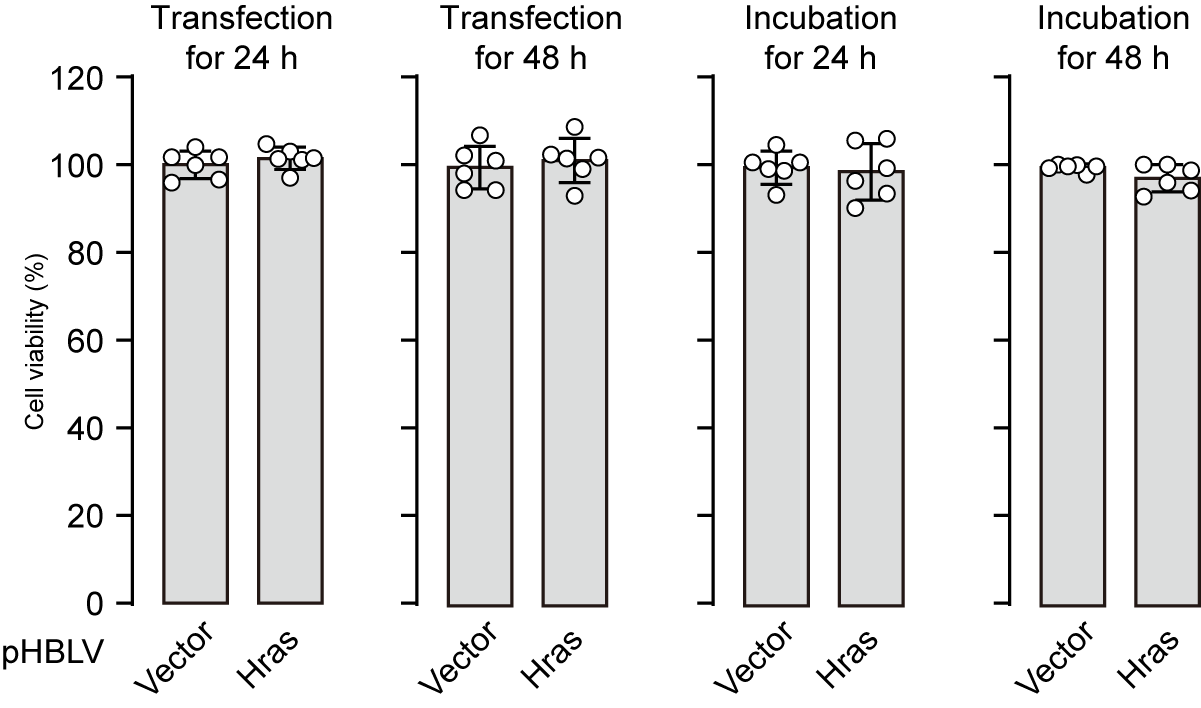
**

**Figure S2.** Hras overexpression had no significant effect on cell survival in HEI-OC1 cells. Cell viability after transfection with pHBLV-Hras and pHBLV-Vector (24 h and 48 h) and the following incubation (24 h and 48 h). All data are presented as the mean ± SD.

**
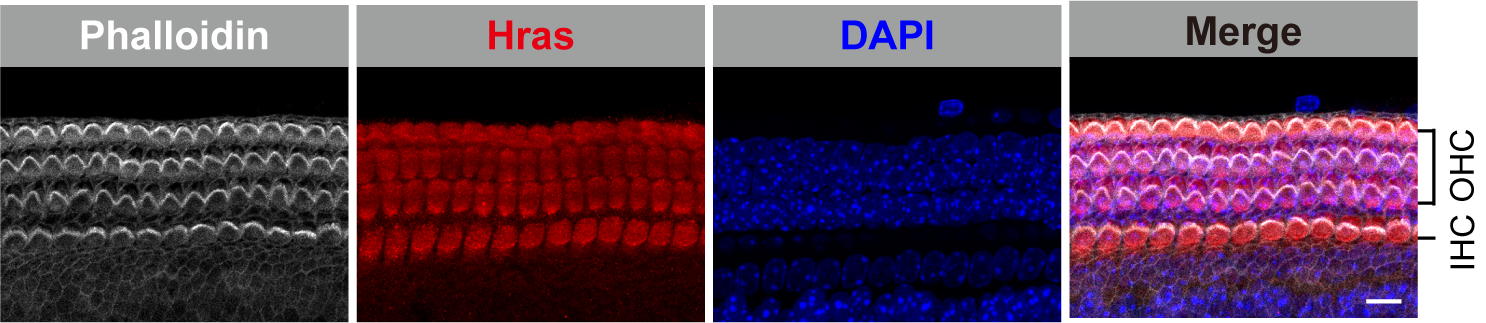
**

**Figure S3.** Representative confocal images showing the self-sustained expression of Hras in neonatal cochleae. Hras (red) in hair cells (Phalloidin stain, white) from the cochlea of an intact P2 C57BL/6 mouse. Cell nuclei were labeled with DAPI (blue). Scale bar, 10 μm. OHC, outer hair cell. IHC, inner hair cell.


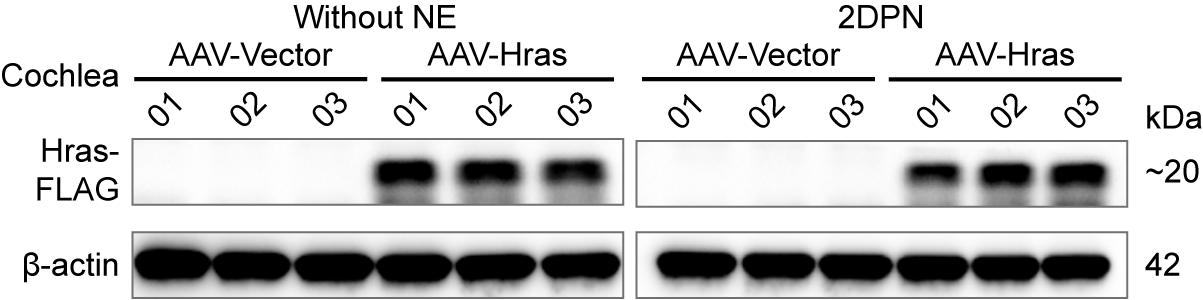


**Figure S4.** Representative Hras-FLAG bands indicating the expression of extrinsic Hras in the AAV-Hras cochlea. NE, noise exposure. 2DPN, 2 days post NE. β-actin was used as the loading control.

**
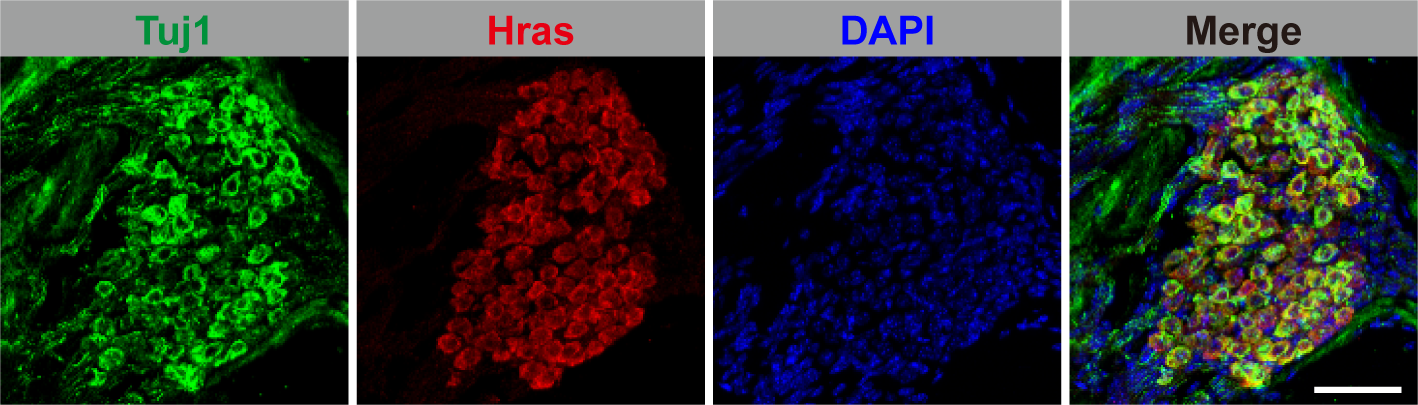
**

**Figure S5.** Confocal images showing the self-sustained expression of Hras in cochlear spiral ganglion neurons (SGNs). Hras (red) in SGNs (Tuj1 stain, green) in cochlear cross-sections from an intact adult C57BL/6 mouse. Cell nuclei were labeled with DAPI (blue). Scale bar, 50 μm.

**
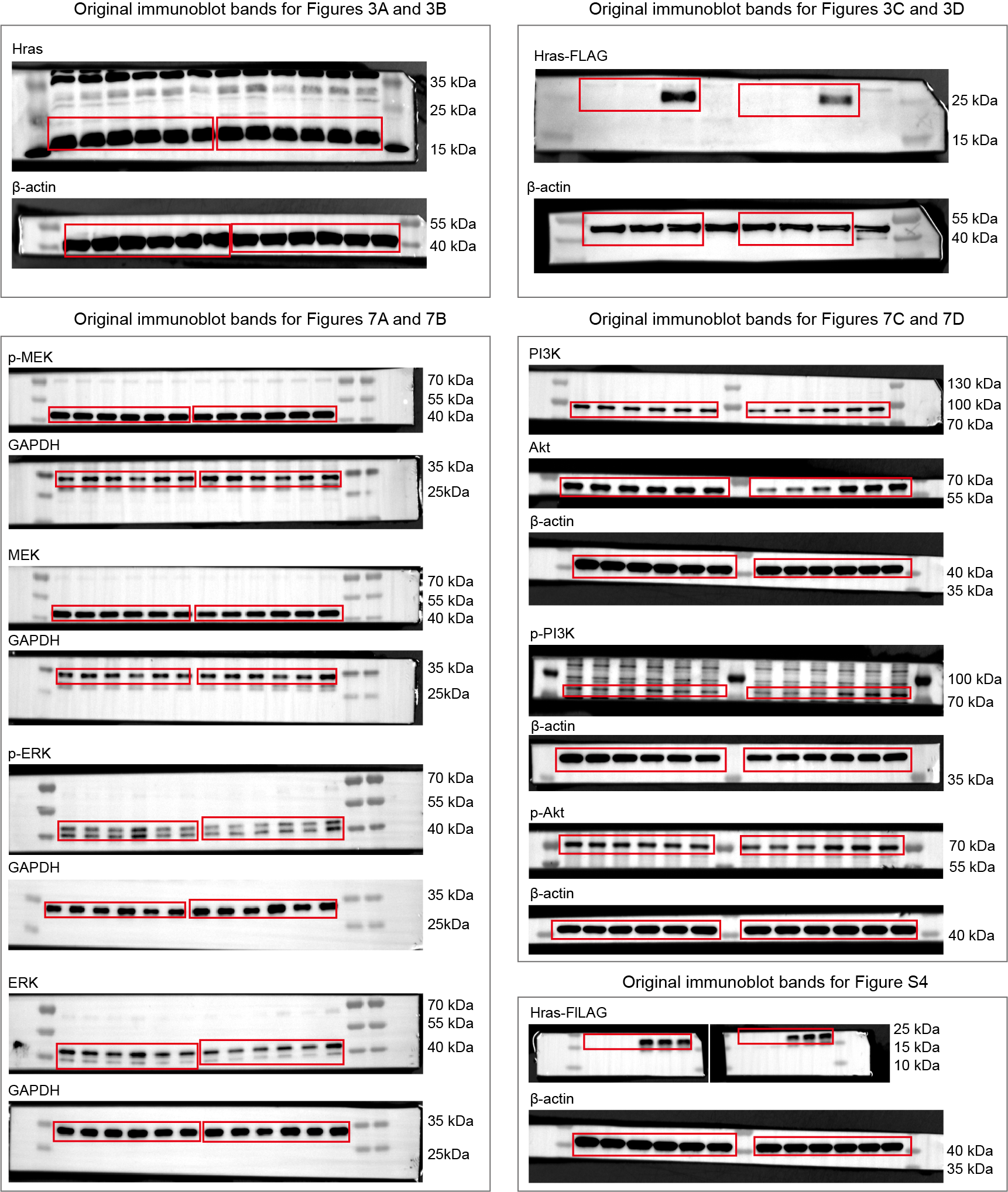
**

**Figure S6.** Original immunoblotting bands.
